# Climate, conflict, and food security: a systematic review of household-level evidence (2020–2025)

**DOI:** 10.1186/s41043-026-01267-0

**Published:** 2026-02-16

**Authors:** Mahlet Degefu Awoke, Tilman Brück

**Affiliations:** 1 The Leibniz Institute of Vegetable and Ornamental Crops (IGZ), Großbeeren, Germany; 2 Humboldt-Universität zu Berlin, Berlin, Germany; 3 ISDC - International Security and Development Center, Berlin, Germany

**Keywords:** Climate crises, Violent conflict, Household food security, Compound crises, Systematic review

## Abstract

Climate and conflict crises increasingly occur together, creating compounded risks for household food security. This review synthesizes evidence from 37 quantitative studies published 2020–2025 on how climate crises (such as drought, storms, or floods), violent conflict (such as war and institutional fragility), and their interactions affect household food security. Most studies examine either climate crises (51%) or conflict crises (38%), while only 11% analyze combined crises. Evidence is geographically concentrated in Sub-Saharan Africa (65%) and relies largely on cross-sectional surveys (68%), limiting insight into longer-term trajectories. Climate crises are measured mainly using meteorological or remote-sensing datasets (42%) while conflict exposure relies predominantly on self-reported data (71%). Only a small number of studies integrate geocoded climate or political violence datasets with household surveys, and few studies estimate interaction or spillover effects. Food security measurement is also narrow, with most studies using access and utilization indicators such as the food consumption score (FCS) (43%), household food insecurity access scale (HFIAS) (35%), or household dietary diversity score (HDDS) (19%). Across studies, climate crises, conflict, and their concurrence are associated with reduced consumption, lower dietary diversity, and greater coping burdens. Impacts vary by household assets, agroecology, and institutional or humanitarian support, highlighting the need for longitudinal and spatially or contextually explicit evidence measuring productive resilience rather than only short-term consumption smoothing.

**Supplementary Information:**

The online version contains supplementary material available at 10.1186/s41043-026-01267-0.

## Introduction

The convergence of climate, political, and economic crises increasingly threatens global food security. Food security exists when “*all people*,* at all times*,* have physical*,* social and economic access to sufficient*,* safe and nutritious food that meets their dietary needs and food preferences for an active and healthy life*” [1, 2]. Despite international commitments to end hunger and despite some improvements in the period between 2000 and 2014 [3], food insecurity has worsened in the period 2015–2023 [4, 5]. In 2023, an estimated 864 million people experienced severe food insecurity, often going an entire day or more without eating, while about 2.33 billion people were moderately or severely food insecure overall [5]. Two of the most pervasive drivers of this reversal are climate change and violent conflict, which individually and jointly undermine food security [1, 6–8]. Climate change intensifies droughts, floods, and heat extremes [1, 9], while violent conflict destroys livelihoods, disrupts markets, and erodes social cohesion [10, 11]. Evidence shows that populations exposed to violent conflict are substantially more likely to experience food insecurity than those in stable settings [12]. Recent work in crisis-affected regions, including Somalia and Ethiopia, shows that climate variability, drought, conflict, and food price shocks simultaneously shape household food consumption, acute malnutrition, and broader food insecurity outcomes [7, 8]. In this study, “crises” are understood as hazards that can cause significant harm to people in a specific locality and period of time [13], including those triggered by climatic shocks and violent conflict. In many regions, these crises now interact, forcing households to navigate simultaneous threats to production, income, and access to food. When these crises occur simultaneously, they create compound crises or polycrises whose impacts may diverge and even exceed those of the sum of the impacts of the individual crises, threatening progress toward Sustainable Development Goal 2 (Zero Hunger for All) and the wider 2030 Agenda [14, 15].

Although research increasingly recognizes that households experience multiple interacting crises, empirical evidence on how climate and conflict jointly affect food security remains limited [12, 15, 16]. Most existing reviews examine either climate–food linkages or conflict–food linkages separately [17, 18], generating important insights on individual pathways but not on how climate and conflict jointly shape food security. In addition, methodological choices (such as study design, crisis exposure variables, and the selection of food-security indicators) vary widely across studies, limiting comparability and the development of generalizable conclusions. Measurement heterogeneity is a key constraint: Food security has commonly been described through four pillars: availability (sufficient supply of food), access (ability to obtain food), utilization (nutritional and biological use of food), and stability (reliability of access over time), with sustainability and agency recently added to reflect equity and decision-making power within the food system [2]. However, individual studies emphasize different pillars and use a variety of survey instruments, recall periods, and classification thresholds. This diversity complicates cross-study comparison and limits the ability to draw generalizable conclusions about how crises shape food security outcomes.

This study surveys these gaps by conducting a systematic review of quantitative, survey-based evidence published in the period 2020–2025 on how climate shocks, violent conflict, and their interaction affect household food security. The review aims to: (1) map where and how crisis–food security relationships are studied, (2) synthesize the direction and nature of reported impacts across standardized food security indicators, and (3) identify conceptual and methodological gaps relevant to compound-crisis or polycrisis research.

By consolidating fragmented empirical evidence, this review advances our understanding of how households experience food insecurity under multiple intersecting shocks. In doing so, it aims to inform future research on food security in polycrisis settings and contribute to analytical discussions that move beyond single-driver perspectives to better reflect the complex interactions between climate shocks and violent conflict shaping contemporary food systems.

## Data and methods

### Search, screening, and inclusion process

We conducted a structured search in Web of Science, ScienceDirect, and PubMed to identify empirical, micro-level studies at the intersection of climate shocks, violent conflict, and household food security. The time window January 2020–April 2025 was chosen to capture the dynamics of overlapping crises and recent advances in measurement while keeping the scope tractable. Our search strings combined three domains: food security, climate shocks, and conflict, and an additional string to capture compound crises. While the core conceptual search terms were applied consistently across databases, the exact search syntax was adapted to accommodate database-specific requirements, including field tags, wildcards, and Boolean operators. The following core final queries illustrate the structure of the search and were applied in Web of Science and PubMed: (1) (“food security” OR “food insecurity” OR “nutrition security”) AND (“climate change” OR “climatic shock*” OR “drought*” OR “flood*” OR “extreme weather”); (2) (“food security” OR “food insecurity” OR “nutrition security”) AND (“conflict*” OR “war” OR “violence” OR “political instabilit*”) NOT (“conflict of interest”); 3) (“food security” OR “food insecurity” OR “nutrition security”) AND (“crisis*” OR “multiple crises*” OR “compound shock*” OR “polycrisis*”). ScienceDirect does not support wildcard operators; therefore, equivalent singular and plural term variants were entered explicitly for that database. The complete database-specific search syntax for all three databases is provided in Appendix 1.

The search returned 3,065 records. Predefined eligibility criteria (Table 1) ensured conceptual and methodological comparability. We included peer-reviewed, quantitative, survey-based studies that reported standardized household food security indicators. We excluded qualitative-only or non-empirical work, papers relying on non-standardized proxies, and studies focused on aggregate food supply rather than micro-level outcomes.

**Table 1:** Eligibility criteria applied in the systematic review

Criterion	Inclusion	Exclusion
Publication type	Peer-reviewed empirical journal articles	Editorials, reviews, blogs, non-empirical work
Language	English	Non-English
Timeframe	2020–2025	Published before 2020
Methodology	Quantitative, survey-based (cross-sectional or panel)	Qualitative-only and review papers
Geography	Global (no restrictions)	–
Shocks addressed	Climate and/or conflict shocks	No climate/conflict component
Food security outcomes	Used standardized food security indicators	Non-standardized measures

**Fig. 1:**
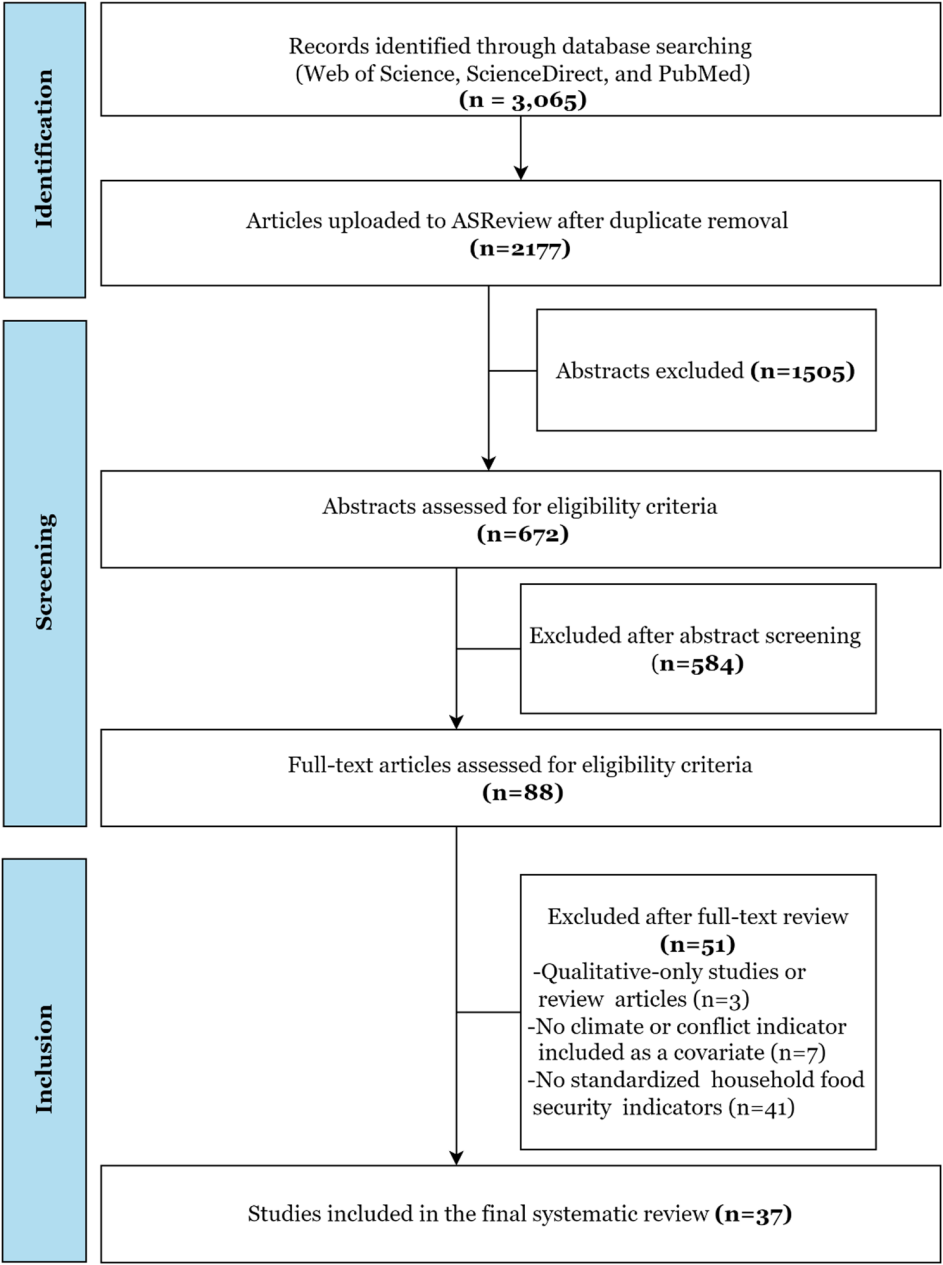
PRISMA flow diagram illustrating the identification, screening, eligibility assessment, and final inclusion of studies in the systematic review

Of these, 88 were accessible and assessed against the inclusion criteria; ultimately, 37 studies met al.l criteria and were included in the synthesis. The entire process followed the PRISMA 2020 [19] guidelines and is summarized in Fig. 1. The completed PRISMA 2020 Checklist is provided in Appendix 2.

We imported bibliographic records into EndNote for metadata management and removed duplicates using Rayyan AI, resulting in 2,177 unique records. We conducted the title and abstract screening in ASReview [20], an open-source active-learning platform operating in a human-in-the-loop mode. Screening was performed by a single reviewer, with the algorithm prioritizing abstracts based on predicted relevance, while the reviewer made all inclusion and exclusion decisions according to predefined eligibility criteria. This hybrid workflow increased screening efficiency, transparency, and reproducibility. Screening stopped once 160 consecutive abstracts were classified as irrelevant, by which point 672 records had been screened, and 89 studies met criteria for full-text assessment. In addition, manual title and abstract screening was conducted to validate the machine-assisted process, which identified two additional potentially relevant articles, yielding 91 full texts for detailed review.

While no formal quality appraisal tool (e.g., JBI, ROBINS-I, or CASP) was used, study quality was assessed through clearly defined and rigorously applied eligibility criteria, standardized outcome measures, and transparent documentation of study design and analytical approaches. This approach aligns with evidence-mapping and knowledge gap map methodologies that prioritize transparency and comparability of evidence [21, 22]. Review procedures were defined a priori and are reported transparently in accordance with the PRISMA 2020 guidelines (Appendix 2).

### Data extraction and synthesis

We extracted data from each of the 37 included studies using a standardized template to ensure consistency and comparability. Extracted variables included bibliographic information (author, year, journal), geographic location, study design (cross-sectional, panel, or repeated cross-sectional), crisis type (climate, conflict, or compound), data sources used to measure crisis exposure, and the specific crisis variables applied. For food security outcomes, all standardized indicators used in each study were recorded, including the indicators applied per crisis type and the reported direction of effect. We classified each indicator according to the Food and Agriculture Organization (FAO) food security pillars, following classifications commonly applied in the empirical food security literature [2, 7, 23–25]. Experiential and economic-access indicators such as the household food insecurity access scale (HFIAS), the food insecurity experience scale (FIES), and the household food security survey module (HFSSM) were categorized under the access dimension [23, 26]. Dietary diversity and consumption-based indicators, including the food consumption score (FCS), household dietary diversity score (HDDS), dietary diversity score (DDS), women’s dietary diversity score (WDDS), and child dietary diversity score (Child DDS), were assigned to the utilization dimension because they reflect diet quality and nutrient adequacy [25, 27]. Indicators linked to household-level food supply were classified under the availability dimension. These include the household food balance model (HFBM), which reflects the sufficiency of food produced or accessible at the household level, and the food expenditure share (FES), which may signal constraints in local food supply when a high proportion of household income is spent on food [4, 27]. Behavioral and coping-capacity indicators, including the reduced coping strategies index (rCSI), the coping strategies index (CSI), and the livelihood coping strategies index (LCSI), were categorized under the stability dimension because they capture how households manage short-term disruptions to food access or availability. The household hunger scale (HHS) was also classified under stability since it reflects acute hunger episodes associated with shocks, although it also overlaps conceptually with access [4, 26]. Several indicators, particularly HDDS, FCS, CSI, LCSI, FES, and HHS, overlap across dimensions because they capture both access-related challenges and short-term stability responses [4, 23–26].

Using the resulting data, we constructed a Knowledge Gap Map (KGM), a structured evidence-mapping tool that visualizes where and how existing research links crises to food security outcomes [21]. Following the approach of evidence gap maps developed by the International Initiative for Impact Evaluation (3ie) [21, 22], the KGM organizes the evidence along two axes: crisis type (climate, conflict, compound) and standardized food security indicators. For each crisis–indicator combination, we coded the reported association (negative, positive, non-significant, or descriptive). We applied interpretation rules consistently across studies; for instance, higher FCS values reflect improved food security, whereas higher HFIAS or FIES scores indicate worsening food insecurity. Consistent with evidence gap map methodologies, the review focuses on mapping the presence, direction, and characteristics of existing evidence rather than grading the certainty or strength of effects, and therefore does not apply formal evidence-grading frameworks such as GRADE.

## Results

### Characteristics of the selected studies

Our final review comprises 37 empirical studies that examine the relationship between climate crises, violent conflict, and household-level food security (Appendix 3). In terms of crisis type, the majority of studies focus on climate-related crises (51%) [28–48], while conflict-related crises account for 38% [49–61]. Only four studies (11%) [62–65] address compound or overlapping climate–conflict crises either directly or indirectly (Table 2).

**Table 2:** Characteristics of included studies

Dimension	Categories	No. of studies	% of total
Crisis type	Climate	19	51%
	Conflict	14	38%
	Climate and conflict	4	11%
	Total	37	100%
Region	Sub-Saharan Africa	24	65%
	South Asia	5	14%
	Middle East & North Africa	4	11%
	Latin America & Caribbean	2	5%
	Southeast Asia	1	3%
	Pacific	1	3%
	Total	37	100%
Study design	Cross-sectional	25	68%
	Repeated cross-section	5	14%
	Panel	7	19%
	Total	37	100%

**Fig. 2:**
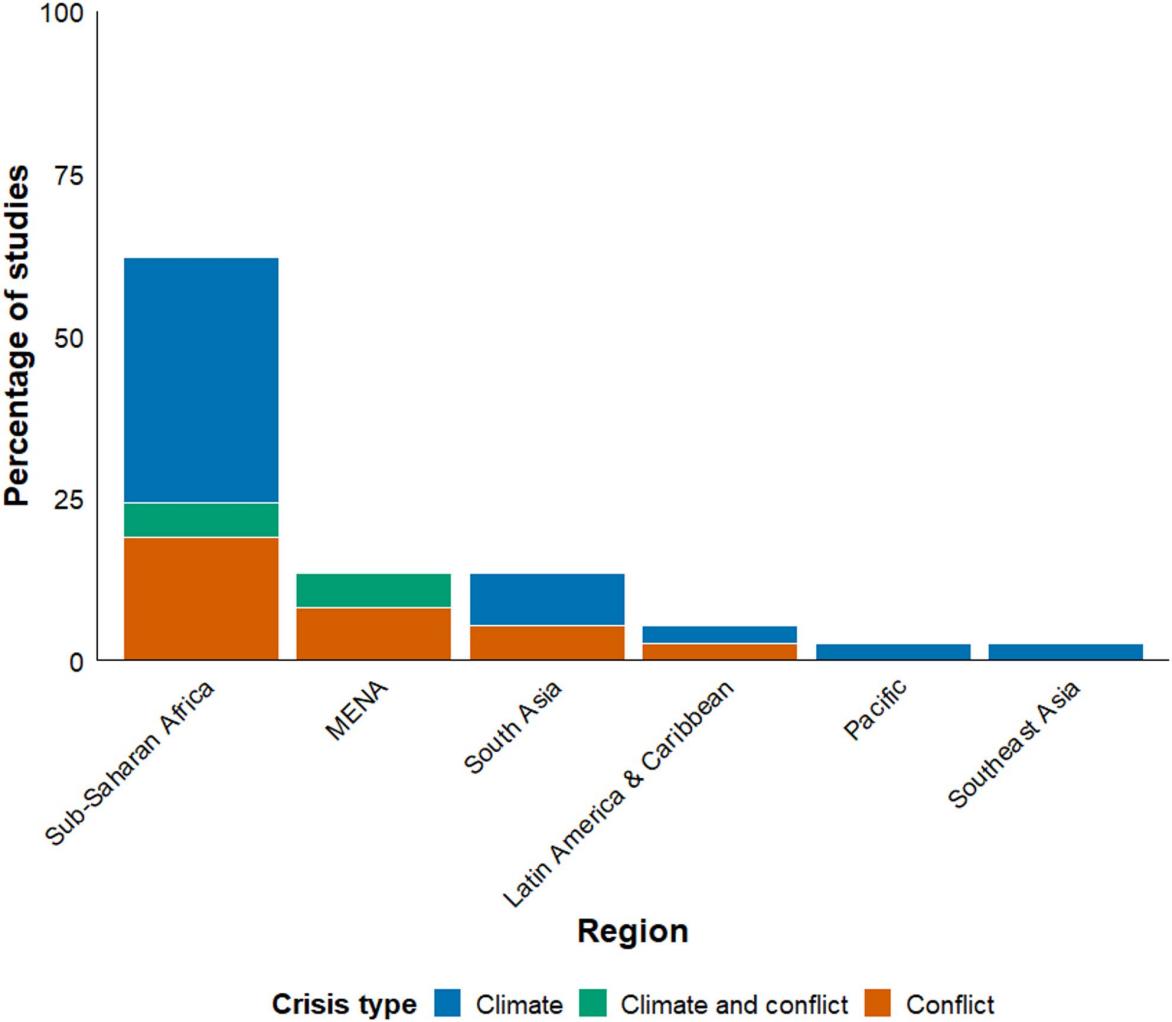
Regional distribution of the included studies, by crisis type (*n* = 37)

Geographically, the evidence base is strongly concentrated in Sub-Saharan Africa (SSA), which accounts for nearly two-thirds of the total studies (65%). Other regions remain comparatively under-represented. Notably, the few studies that analyze compound crises are limited to SSA and the Middle East and North Africa (MENA). Methodologically, the literature relies heavily on cross-sectional surveys (68%), with fewer panel (19%) and repeated cross-sectional (14%) designs. Overall, the distribution reveals a geographically and methodologically uneven evidence base: research is concentrated in African settings and cross-sectional surveys, while compound crises and longitudinal analyses are scarce. These asymmetries shape what is currently known about the joint influence of climate and conflict on household food security. Figure 2 illustrates the regional distribution of studies by crisis type, highlighting the pronounced dominance of SSA.

### Crisis data sources

The reviewed studies differ substantially in how they capture exposure to climate and conflict crises. Some draw on objective environmental or administrative datasets, while others rely on household-level perceptions or retrospective recall. For comparability, we grouped crisis data source into four main categories: (i) geocoded conflict datasets such as the Armed Conflict Location and Event Data (ACLED) or the Uppsala Conflict Data Program (UCDP); (ii) standardized meteorological datasets including the Climate Hazards Group InfraRed Precipitation with Station Data (CHIRPS), CHIRTSmax, or TerraClimate; (iii) humanitarian monitoring databases such as the Displacement Tracking Matrix (DTM); and (iv) household survey–based reports of crises such as droughts, floods, displacement, or violence.

As shown in Table 3, climate-related studies rely both on objective and perception-based sources to a similar extent. About 42% use meteorological or gridded datasets (26% and 16%, respectively), while roughly one-quarter rely on household reports of rainfall anomalies or extreme events. Another 26% adopt hybrid approaches, combining survey data with meteorological or humanitarian records to triangulate exposure measures (Appendix 4).

**Table 3:** Crisis primary data sources

Crisis type	Data source type	Number of studies	% of studies	Example of data sources
Climate	Gridded / remote-sensing climate datasets	3	16	CHIRPS/CHIRTS, TerraClimate
	National meteorological & statistical records	5	26	National yearbooks, met-station drought/flood data
	Survey-based	6	32	Perceived drought, flood, or rainfall variability
	Hybrid (survey + gridded / meteorological/humanitarian database)	5	26	Mixed approaches (e.g., CHIRPS + survey)
	Total	19	100%	
Conflict	Geocoded conflict datasets (event-based)	3	21	ACLED or UCDP
	Survey-based	10	71	Self-reported violence, displacement, or asset loss
	Hybrid (Geocoded + Survey-based)	1	7	ACLED + LSMS-ISA combined approach
	Total	14	100%	
Climate and Conflict	Humanitarian monitoring databases	1	25	Displacement Tracking Matrix
	Survey-based	3	75	Perceived or reported dual exposure (climate + conflict)
	Total	4	100%	

Abbreviations: CHIRPS = Climate Hazards Group InfraRed Precipitation with Station data; CHIRTS = Climate Hazards Group InfraRed Temperature with Station data; TerraClimate = TerraClimate global gridded climate dataset; ACLED = Armed Conflict Location & Event Data Project; UCDP = Uppsala Conflict Data Program; LSMS-ISA = Living Standards Measurement Study – Integrated Surveys on Agriculture; DTM = Displacement Tracking Matrix

### Crisis exposure measurement

**Fig. 3:**
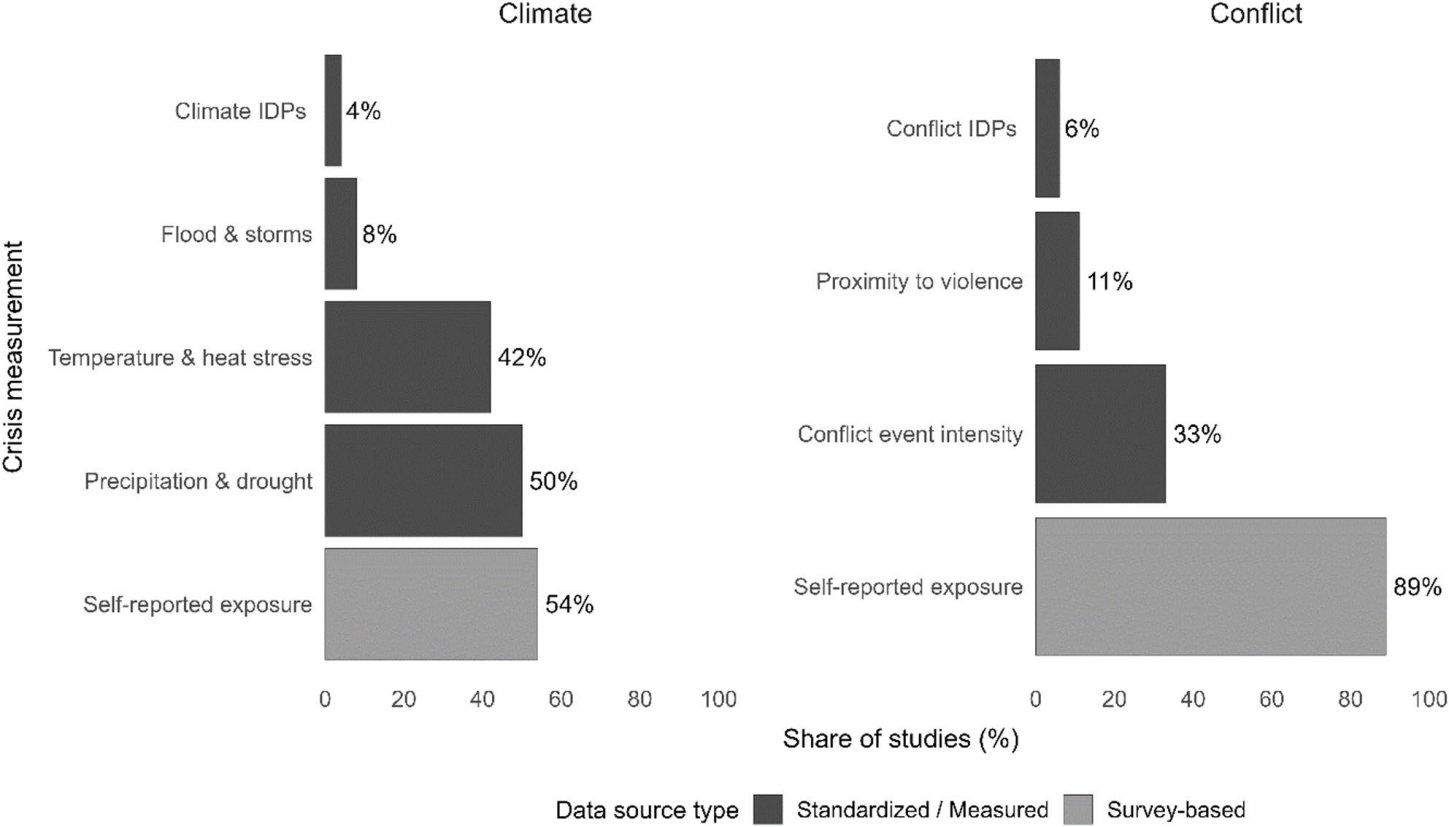
Crisis exposure variables by data source (climate: *n* = 23; conflict: *n* = 18). *Note: Percentages exceed 100% because a single study may use multiple indicators. IDP=Internally Displaced Persons*

Building on the data sources summarized above, the reviewed studies also differ in how climate and conflict crises were operationalized as exposure variables (Fig. 3). Compound-crisis studies are counted in both groups, which results in 23 climate-related and 18 conflict-related observations, even though the review comprises only 37 studies. Several studies include multiple crisis variables within the same design (e.g., drought and displacement), allowing a single study to contribute multiple exposure observations. Consequently, the number of exposure variables exceeds the number of studies, and percentages in the figure sum to more than 100% (Appendix 3). Note that compound events may also exist within a category. For example, a climate shock may entail both hot and dry weather, where each component (heat and lack of precipitation) has distinct impacts, with a compound event (hot and dry) possibly having additional impacts [37]. However, at a higher level of aggregation, we focus here on compound events consisting of climate and conflict components.

Among climate-focused studies, more than half (54%) measure precipitation and drought anomalies, often using standardized indices such as CHIRPS/CHIRTS, while 42% capture temperature or heat-stress variation. Few studies quantify floods or storms (8%) or use internally displaced persons (IDPs) influx (4%) as indirect stress indicators. Notably, more than half (54%) rely on self-reported weather shocks, reflecting perceived rainfall irregularity or drought conditions. In conflict-related analyses, 89% of studies use self-reported exposure to violence, insecurity, or displacement, while 33% employ event-based intensity measures, 11% use proximity-to-violence metrics, and 6% include IDP inflows as proxies of conflict pressure. Taken together, these patterns reveal a consistent asymmetry: climate crises are more often measured through standardized datasets, whereas conflict exposure relies primarily on self-reported survey-based data.

### Food security indicator prevalence and knowledge-gap map (KGM) results

Across the reviewed studies, food security is measured using a narrow set of indicators focused on utilization and access (Table 4). The food consumption score (FCS) is used most frequently (43%), followed by the HFIAS (35%), while HDDS and FIES each appear in 19% of studies, and the rCSI in 14%. The remaining indicators (DDS, CSI, LCSI, HFSSM, WDDS, Child DDS, FES, HFBM, HHS) occur only sporadically (≤ 5%). Although 40% of studies apply more than one indicator, most measurements still reflect food access, availability, and utilization outcomes rather than stability, sustainability, or agency (Appendix 5).

**Fig. 4:**
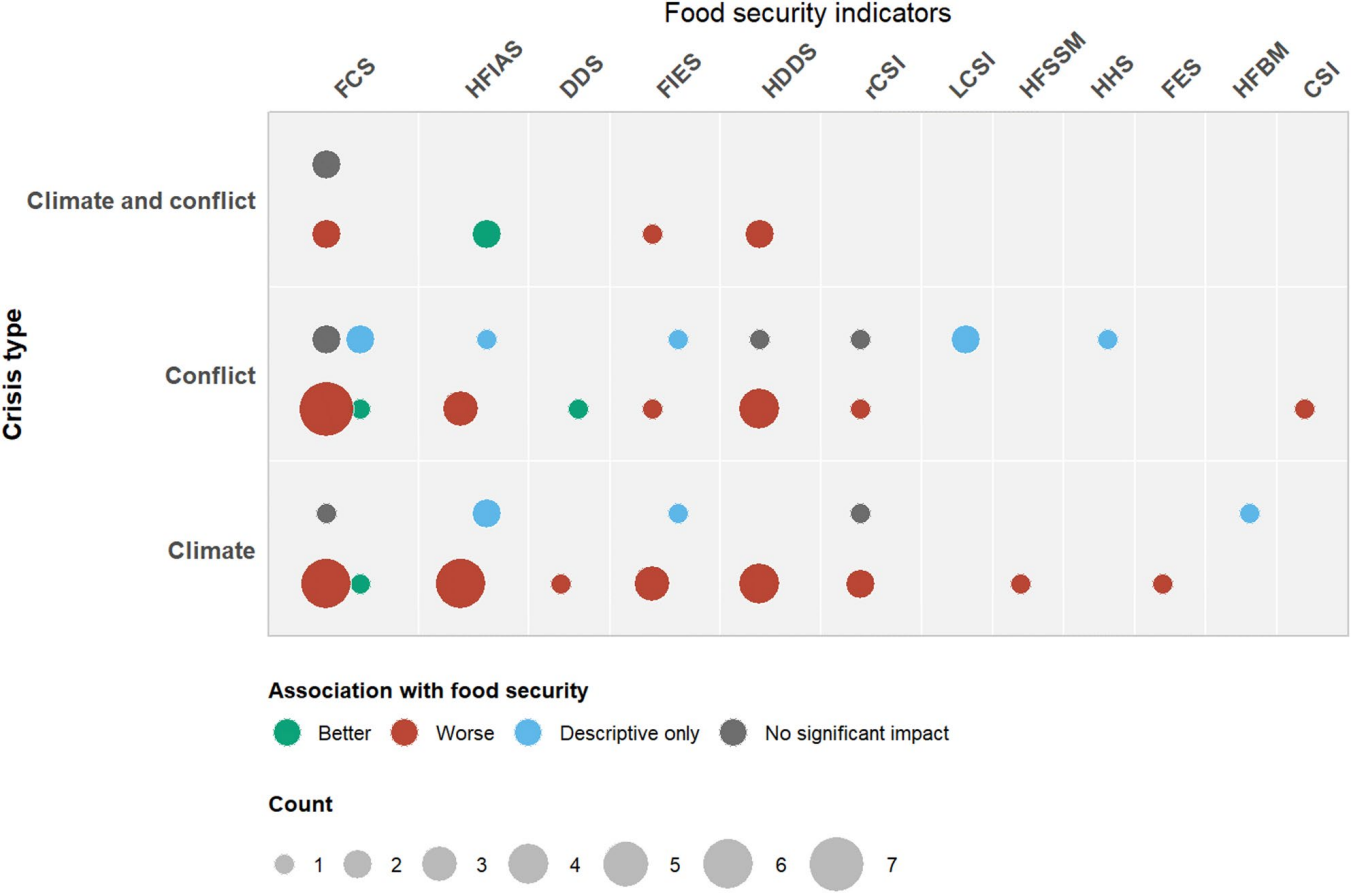
Knowledge Gap Map of associations between crisis types and food security indicators. Bubble colour shows the direction of association, and bubble size reflects the number of reported crisis–indicator associations. Because indicators differ in meaning (for example, higher FCS and HDDS indicate better food security, while higher HFIAS and rCSI indicate worse food security), the direction of association was interpreted according to each indicator’s definition. Associations are therefore summarized as “better” for higher food security outcomes, “worse” for lower food security outcomes, “no significant impact” for statistically insignificant results, and “descriptive only” for studies without inferential analysis

The knowledge gap map (Fig. 4) reveals strong consistency in the direction of empirical evidence: most studies find that both climate and conflict-related crises are associated with adverse food security outcomes.

Climate studies consistently find that droughts, rainfall variability, temperature extremes, and floods reduce household food security. Impacts are evident through declines in consumption and dietary diversity (lower FCS and HDDS) and an increase in access-related food insecurity (higher HFIAS, FIES, or rCSI). For example, droughts in Senegal heighten coping burdens and reduce food consumption [39]. While in Ethiopia and Ghana, rainfall variability lowers food consumption scores and increases access-related insecurity [32, 37, 40, 45]. In Bangladesh, excessive rainfall and flooding reduce agricultural income and, in turn, worsen household food security, underscoring the need for site-specific adaptation support [28, 41].

Conflict-related studies reveal similar deterioration in food security but through different transmission pathways, primarily displacement, loss of assets, market disruption, and reduced access to agricultural land. Several of the reviewed conflict studies show that food insecurity increases with conflict exposure (higher HFIAS/FIES/rCSI) [39, 49, 57, 58], and dietary diversity and consumption decline (lower HDDS/FCS) [39, 52, 53, 55, 62]. However, humanitarian assistance can buffer these effects. In Syria, despite high insecurity, humanitarian distributions temporarily improve FCS and DDS, while households simultaneously report elevated coping burdens (rCSI), revealing that consumption gains can mask underlying stress [51].

Among the four studies [62–65] that include both climate and conflict variables, only two examine the impact of interactions between crises [64, 65]. These studies explicitly test compounding effects rather than treating climate and conflict independently. The first, focusing on Ethiopia and Malawi, reports that conflict significantly reduces household food consumption, and that this effect becomes even stronger when droughts or floods occur in the same period, showing the amplifying nature of simultaneous crises [64]. The second study constructs a vulnerability index that combines three stressors: climate crises, conflict, and environmental degradation, and finds that exposure to multiple crises substantially increases the likelihood of food insecurity [65]. The remaining two studies [62, 63] include both climate and conflict variables in the analysis but evaluate their effects separately, without estimating interaction terms.

**Table 4:** Food security indicator prevalence

Food security indicator	Climate	Conflict	Climate and Conflict	Total no of studies using the indicator	%	Represented food security pillars
FCS	5	9	2	16	43%	Access, utilization & availability
HFIAS	8	4	1	13	35%	Access & availability
HDDS	4	2	1	7	19%	Utilization, access & availability
FIES	4	2	1	7	19%	Access & availability
rCSI	3	2	0	5	14%	Stability & access
DDS	1	1	0	2	5%	Utilization, access & availability
LCSI	0	2	0	2	5%	Stability, availability & access
CSI	0	1	0	1	3%	Stability, availability & access
Child DDS	1	0	0	1	3%	Utilization, availability& access
FES	1	0	0	1	3%	Access & availability
HFBM	1	0	0	1	3%	Availability
HFSSM	1	0	0	1	3%	Access & availability
HHS	0	1	0	1	3%	Access & availability
WDDS	1	0	0	1	3%	Utilization, availability & access

Note: Percentages exceed 100% because a single study may use multiple indicators (*N* = 37)

Abbreviations: FCS = Food Consumption Score; HFIAS = Household Food Insecurity Access Scale; HDDS = Household Dietary Diversity Score; FIES = Food Insecurity Experience Scale; rCSI = reduced Coping Strategies Index; DDS = Dietary Diversity Score; LCSI = Livelihood Coping Strategies Index; CSI = Coping Strategies Index; Child DDS = Child Dietary Diversity Score; FES = Food Expenditure Share; HFBM = Household Food Balance Model; HFSSM = Household Food Security Survey Module; HHS = Household Hunger Scale; WDDS = Women’s Dietary Diversity Score

Very few studies (six) report no significant association or mixed findings between crises and food security. In some cases, non-significant results arise where baseline deprivation is so severe that there is little variation to detect, and in others where humanitarian assistance smooths short-term consumption. In Yemen, for example, conflict-related port closures and fuel shortages produced uniformly poor food access across regions, leaving too little variation for detecting a relationship between conflict violence and FCS; for the rCSI, households had already exhausted coping options early in the conflict, creating a plateau that prevented further increases despite worsening conditions [61]. In rural Syria, households facing insecurity-related land access constraints show higher FCS and DDS values, not because they are food secure, but because humanitarian assistance elevates short-term consumption. Yet these same households display elevated coping burdens, indicating that improved consumption can coexist with underlying stress and asset erosion [51].

Such mixed patterns also emerge in climate-related studies. In Uganda, rainfall variability produces both positive and negative effects on food security depending on the agroecological context: increased rainfall improves FCS in semi-arid zones, while excessive rainfall and flooding reduce food security in high-rainfall areas [41]. The same study finds that agricultural adaptation strategies, such as crop diversification, improved varieties, fertilizer use, and mixed crop–livestock systems, significantly increase the probability of acceptable food consumption scores, showing that productive capacity buffers households against climate variability. This highlights that climate impacts are not uniformly negative; outcomes are mediated by agroecology and adaptation options.

Context also shapes conflict-related outcomes. For instance, a study from South Sudan shows that humanitarian assistance improves food consumption scores within protection camps, while rural households experience deeper food insecurity because insecurity restricts access to markets and aid distribution [59]. Households in both settings (protection camps and rural areas) resort to irreversible coping strategies, such as asset liquidation and meal reduction, signaling persistent livelihood stress despite temporary consumption support. Although humanitarian assistance significantly reduces the likelihood of severe food insecurity, it does not improve dietary diversity, indicating that emergency aid mitigates acute deprivation without enhancing nutritional adequacy or rebuilding resilience.

Only one study examines the spatial spillover effects of conflict [54]. The study analyzes violent conflict in Uganda, Ethiopia, and Malawi and shows that conflict reduces food security not only where violence occurs, but also in neighboring districts through market disruptions, displacement flows, and insecurity spillovers. This highlights an analytical gap: most studies treat households as isolated units, even though crises propagate through markets, mobility, and networks. Future research could model and study these connections and spillovers more explicitly.

Taken together, these findings show that climate, conflict, and their interactions influence food security through similar mediating mechanisms: income loss, reduced agricultural production, market inaccessibility, displacement, and asset depletion. Remittances and humanitarian assistance can buffer short-term effects, but rarely restore productive capacity or improve dietary quality. This pattern reveals structural vulnerability, where households cycle through crises without fully recovering.

As reflected in the KGM (Fig. 4), the evidence base is heavily concentrated on availability, access, and utilization-focused indicators such as FCS, HDDS, HFIAS, and FIES; in contrast, indicators capturing stability, sustainability, or agency remain largely absent. While nutrition-sensitive indicators such as WDDS were mentioned in one study, they were not linked to any climate or conflict indicator [35]. Inclusion of indicators in the KGM required that food security indicators be explicitly linked, either descriptively or statistically, to climate or conflict crises. Indicators such as LCSI, CSI, HHS, and HFBM met this criterion and were therefore included, even when the linkage was reported only once. As a result, WDDS could not be positioned within a crisis–food security relationship and was excluded from the KGM. The current evidence can tell us whether households eat, but less often whether they eat adequately, consistently, or with autonomy.

## Discussion

Our review reveals a consistent pattern: climate crises, violent conflict, and their interactions are associated with deteriorations in household food security, reflected in lower food consumption, reduced dietary diversity, and increased reliance on adverse coping strategies. Beyond this, the evidence base reveals structural imbalances in where studies are conducted, how crises are measured, and how food security is conceptualized. Nearly two-thirds of studies take place in Sub-Saharan Africa, and most rely on cross-sectional household surveys, leaving major crisis-affected regions such as the Middle East, Latin America, and South Asia comparatively understudied. We identify five implications from our review. The strong geographic concentration of the evidence base has important implications for the interpretation and generalizability of these findings.

First, crises are measured in heterogeneous and, at times, non-standard ways. Climate crises are generally quantified using meteorological or remote-sensing products, whereas conflict exposure is predominantly captured through household self-reports of violence, displacement, or asset loss. These approaches generate fundamentally different types of information and reduce the comparability of findings. Gridded datasets provide spatial and temporal precision, yet they may overlook how different households in the same location experience climate variability differently through disrupted production or income pathways. In contrast, perception-based or self-reported measures capture lived realities, including non-fatal insecurity, but they are vulnerable to recall and salience biases [66]. Very few studies integrate geocoded conflict-event data with climate datasets and household survey information, even though such integration would allow spatially explicit analysis of how crises unfold, interact, and spread across space. Future research could work toward more consistent and mixed measurement approaches that combine geocoded crisis data with household-survey information to improve comparability across studies.

Second, the dominant reliance on cross-sectional survey designs limits the ability to observe recovery, persistence, or cumulative effects of repeated shocks over time. Crises arise dynamically, their interactions are dynamic, and so are their impacts. Hence, the field needs much more focus on the use of panel data (preferably long-term panel data) to understand better the mechanisms at stake. Future research would benefit from expanded use of longitudinal data linked with crisis-exposure measures to trace how impacts evolve and accumulate over time.

Third, most studies examine single crises in isolation. Only a small number include both climate and conflict exposure in the same model, and interaction terms are rarely estimated. Yet evidence from related fields shows that overlapping crises can amplify vulnerability in non-linear ways [14, 15]. Similarly, spatial spillovers are almost entirely absent: only one study estimated spillover effects, even though market disruptions, displacement, and humanitarian access inherently cross administrative boundaries. Future research should explicitly examine joint exposure, estimate interaction effects, and incorporate spatial analysis to capture how crises reinforce each other and spread across space.

Fourth, our review also highlights substantial heterogeneity in outcomes. Similar crises do not produce uniform effects on food security. Instead, outcomes depend on households’ adaptation capacity, market access, and the presence of humanitarian or institutional support. Consistent with previous evidence, households with productive assets, such as crop diversification, improved varieties, and mixed crop–livestock systems, maintain consumption more effectively during climate crises [67]. In conflict settings, humanitarian assistance can temporarily stabilize consumption but rarely rebuild productive capacity, leaving households vulnerable when support is interrupted. This suggests that it is not only crises that create vulnerability - crises also expose existing structural inequalities in the ability to cope and recover.

Fifth, the conceptualization of food security in this field remains narrow. Most studies rely on consumption- or access-based indicators (FCS, HFIAS, HDDS, FIES). These instruments capture short-term outcomes but do not account for stability, sustainability, or agency, dimensions emphasized in current food security frameworks [2]. As a result, the existing evidence can tell us whether households have enough to eat, but not whether they eat adequately, consistently, or with the autonomy to make choices. Overall, these findings show that food insecurity in crisis-affected settings is shaped not only by exposure to crises, but also by households’ capacity to adapt and by institutions’ ability to reach them during crisis. Future research should broaden the set of indicators used, incorporating dimensions such as sustainability and agency to reflect current food-security frameworks.

Our review is limited by its focus on peer-reviewed, quantitative, English-language studies. While this approach ensured methodological consistency and comparability across studies, it excludes insights from qualitative work, policy reports, and other grey literature. In addition, the strong geographic concentration of the reviewed studies reflects the current distribution of empirical research rather than a deliberate restriction and limits the extent to which the findings can be generalized beyond the contexts represented in the literature. Consequently, the relevance of the findings for regions such as Asia, Latin America, or the Middle East should be interpreted with caution. The emphasis on standardized, household-level indicators enhanced coherence but narrowed the conceptual scope of food security measurement. Restricting the timeframe to 2020–2025 allowed timely insights but excluded longer historical trajectories. Future reviews could build on this foundation by integrating mixed-methods evidence and grey-literature sources to capture a more comprehensive and nuanced understanding of how crisis interactions shape food security.

## Conclusions

Our review demonstrates that climate crises, violent conflict, and their interactions undermine household food security, most clearly through reduced consumption, lower dietary diversity, and greater reliance on coping strategies. However, the severity of these impacts varies. Outcomes are shaped not only by the crisis itself but also by the assets, livelihood strategies, and institutional support households can draw upon. Where productive resources or assistance are available, declines in food security are moderated. Where they are absent, crises quickly translate into severe and prolonged insecurity. Critical gaps remain in the evidence base. Most studies are concentrated in Sub-Saharan Africa and rely on cross-sectional data, which limits our understanding of recovery or cumulative effects over time. Very few studies examine situations in which climate and conflict pressures co-occur, and spatial dynamics are rarely considered, even though markets, displacement, and humanitarian access extend beyond individual survey locations.

From our analysis, three implications are relevant for policy and practice. First, adaptation must be context-specific. The review shows that similar crises do not produce similar outcomes; food security impacts vary with agroecological conditions, livelihood strategies, and institutional support. Second, households face both short-term and longer-term effects, suggesting that crisis responses may need to balance immediate consumption support with efforts that help restore productive capacity, household assets, and diversified production systems in the triple nexus. Third, several studies highlight the importance of functioning markets and timely input delivery. Strengthening market access and access to seeds, fertilizers, and improved varieties appears to reduce vulnerability to both climate and conflict crises by stabilizing production and lowering exposure to localized crises.

In conclusion, our findings suggest a necessary shift in focus, both in policy and in research. Reducing food insecurity in crisis-affected contexts requires investment in conditions that enable households to withstand and recover from crises, rather than policies that stabilize only short-term consumption. Future research would benefit from linking geospatial and longitudinal data and from using food security metrics that capture not only consumption but also stability, sustainability, and agency. Without such shifts in future research, policy may continue to respond to food insecurity as an emergency outcome rather than as a consequence of structural vulnerability.

## Supplementary Information

Below is the link to the electronic supplementary material.

Supplementary Material 1

Supplementary Material 2

Supplementary Material 3

Supplementary Material 4

Supplementary Material 5

Supplementary Material 6

## Data Availability

All data generated or analyzed in this study are provided within the article and its supplementary information. Further details are available from the corresponding author upon reasonable request.
