# Supplementary material for: Climate, conflict, and food security: a systematic review of household-level evidence (2020–2025)
Source: J Health Popul Nutr. 2026 Feb 16;45:70. doi: 10.1186/s41043-026-01267-0 (PMC12918621; doi:10.1186/s41043-026-01267-0)
Supplement: Supplementary file 6 — Supplementary Material 6 [file 41043_2026_1267_MOESM6_ESM.pdf]

Search syntax

Web of Science and PubMed

1. ("food security" OR "food insecurity" OR "nutrition security") AND ("climate change" OR "climatic shock*" OR "drought*" OR "flood*" OR "extreme weather");
2. ("food security" OR "food insecurity" OR "nutrition security") AND ("conflict*" OR "war" OR "violence" OR "political instabilit*") NOT ("conflict of interest");
3. ("food security" OR "food insecurity" OR "nutrition security") AND ("crisis*" OR "multiple crises*" OR "compound shock*" OR "polycrisis*")

ScienceDirect

1) ("food security" OR "food insecurity" OR "nutrition security") AND ("climate change" OR "climatic shock" OR "climatic shocks" OR "drought" OR "droughts" OR "flood" OR "floods" OR "extreme weather")
2) ("food security" OR "food insecurity" OR "nutrition security") AND ("conflict" OR "conflicts" OR "war" OR "wars" OR "violence" OR "political instability" OR "political instabilities") NOT ("conflict of interest")
3) ("food security" OR "food insecurity" OR "nutrition security") AND ("crisis" OR "crises" OR "multiple crises" OR "multiple crisis" OR "compound shock" OR "compound shocks" OR "polycrisis")
